# Miscellanies

**Published:** 1835-01-01

**Authors:** 


### MISCELLANIES.

### CASE OF HEPATIZED SPLEEN. By JAMES EDWARD, Surgeon, Forfar.

WE are indebted to Mr. Edward, for several interesting facts. The following has lately occurred to his observation, and is contained in a letter which he has addressed to us.

"The subject of this case was a very laborious tradesman, of temperate habits, forty years of age. He was suddenly seized with acute pain in the region of the spleen, immediately on the inside of the left margin of the ribs, which was increased by pressure, and extended up to the shoulder of the same side. It was attended with a full, quick pulse, heat, thirst, and other symptoms of inflammatory fever. By venesection both local and general, with other antiphlogistic remedies, these symptoms were for a time alleviated. The pain in the side was a pretty constant symptom during the first twenty months of this disease; at the end of which a tumor of an oblong figure could be distinctly felt through the parietes of the abdomen in the left side. This tumor gradually increased till it occupied the whole of that region. For several weeks previous to his death, which happened in November, 1833, after having been ill rather more than three years, his mental faculties were so completely destroyed that he did not recognize his most intimate friends.

On a *post-mortem* examination, which was held twenty-four hours after death, the abdomen having been opened, an enlarged spleen was removed, of an oblong figure, convex externally, and concave internally, the substance of which was similar to that of the liver; it weighed six pounds, eight ounces. With the exception of a small cyst in the left kidney, the rest of the abdominal and thoracic viscera seemed to have been perfectly sound. The brain was not examined.

Forfar, Dec. 16th, 1834."

### MEDICAL EDUCATION AND ORGANIZATION.

We request the attention of our brethren to the following system of medical polity acted on in the Austrian empire, and from which some valuable materials may be drawn for the construction of the social edifice that may be expected soon to rise in this country. We certainly should not be much inclined to go to the banks of the Danube for political tenets or doctrines; but science, and especially medical science, is the growth of no country—and a Metternich might probably legislate as wisely in physic as a Melbourne. We shall introduce the Austrian code of medical police without further preface.

### *Present System of Medical Education, and the Arrangement of the Medical Profession, in the Austrian States.*

The laws by which the profession in Austria is governed, were enacted in the reigns of Maria Theresa, of Joseph II., and of the Emperor Francis I. The object is the improvement of medical education in the schools, the supplying of suitable practitioners for the people, and the regulation of the *pharmaciens*, who alone are permitted to deal in drugs and medicines. They further contain clauses of great severity for the checking of charlatanism; and in order that the magistrates may be fully acquainted with every thing relating to hygiene and medical police, appointments are provided in the several districts for a body of well-instructed and experienced medical persons, specially entrusted with this duty.

#### I. SYSTEM OF EDUCATION.

*There is no distinction made, in the Austrian universities, between the education of physicians and that of surgeons. The students destined for either pursuit must attend the courses of both branches of the healing art, and not until they have com-*

*pleted their curriculum are they allowed to choose which they shall practise: but they may practise both if they choose, and be found properly qualified.*

The qualification for a doctor of medicine or surgery, consists in a *five years' attendance of lectures in some national university;* the three first years being devoted to the study of the collateral sciences and the theoretical parts of medicine, and the last two being employed in special therapeutics and practice, at the bedside of the patient. The following is the order in which the several branches are studied:—

| | | |
|---|---|---|
| First Year ... | 1st Semestre. | A general introductory course of medicine and surgery.<br>A course of anatomy.<br>A course of special natural history. |
| | 2d Semestre. | The courses of anatomy and natural history repeated.<br>A course of botany. |
| Second Year. | 1st Semestre. | Anatomy and physiology of a more advanced character.<br>General chemistry. |
| | 2d Semestre. | Anatomy and physiology continued.<br>Pharmacy and animal chemistry. |
| Third Year .. | 1st Semestre. | 1. General pathology (etiology, semeiology, and general therapeutics.)<br>2. Materia medica and chirurgica, dietetics, and art of prescribing.<br>3. Theoretical surgery (general and special pathology of surgical disorders.)<br>Midwifery. |
| | 2d Semestre. | Courses 1, 2, 3, preceding, continued.<br>Bandages and surgical instruments, from June till the end of the medical year. |
| Fourth Year. | 1st Semestre. | 1. Special therapeutics of internal maladies.<br>2. Internal clinique. |
| | 2d Semestre. | 1 and 2 preceding continued.<br>Veterinary medicine. |
| Fifth Year .. | 1st Semestre. | 1 and 2 of preceding year continued.<br>Forensic medicine. |
| | 2d Semestre. | 1 and 2 continued.<br>Medical police. |

It is to be observed, with reference to clinical instruction, that, both in Austria and Prussia, students who have completed their theoretical courses are divided into practising pupils and assistants; the former being entrusted with the treatment of a certain number of patients, whom they visit under the inspection of the clinical professor. If they acquit themselves well, he does not interfere; if not, he instructs them in the questions which they ought to put. After each visit, the professor interrogates the pupil as to the class, order, and species of the malady, the prognosis, and the indications. If the pupil be right, he is requested to prescribe aloud. An assistant is attached to each practising pupil, who goes round with him, and in the course of six months becomes a practitioner himself.

The lectures on physiology, pathology, materia medica, and special therapeutics, as well as the clinical remarks at the patient's bed side, are delivered in Latin; in all the other courses the German is the language employed. In Hungary, Poland, and Italy, the language of each country respectively is used.

*Previous to admission to the medical schools, the pupil must produce a certificate of having attended a three years' course of humanity in some national school; and pupils are arranged, in general, in three classes; the first consisting of those who have answered best, and obtained the title of* eminent, *the others according to their respective merits.* Diligence and moral conduct are high recommendations in the certificate for admission; in fact, the law expressly declares that this must be seriously attended to, in order to exclude, as much as possible, from the study of an art so important and difficult as that of medicine, all those who are not more or less distinguished by their attainments and good conduct.

Matriculation is not attended with any expense.

*Students are forbidden to smoke cigars, or to frequent drinking-houses.*

No student can advance to a higher class without having attended that immediately preceding it; and he must pass an examination. If his answering be but second-rate, he must go through his last courses again; and if upon another trial he be found deficient, his name is erased from the list of medical students, and he is precluded from entering any other national university.

Every professor is bound to examine his class once a week publicly, for at least half an hour. The results he must note down, for the better arrangement of the classes. At the end of every semestre, the pupils are examined in their previous courses; the first examination takes place in the latter part of March, the second towards the end of August; the particular day and hour being announced a month previously. The director and a commissioner of instruction are obliged to attend at these trials; and the professors are enjoined by the law to be as strict as possible, and not to allow themselves to be carried away by an ill-judged indulgence. It is during the first year that they are expected to be most severe, in order to get a timely riddance of those students who are dull or negligent, and to secure the state against the danger of having ignorant physicians or surgeons admitted to practice. In the certificates given on these occasions, not only the abilities of the pupil are set forth, but his moral conduct is noted.

The fees paid by each student amount to 30 florins (about 3l.) a year—3 florins a month. These charges go chiefly to the support of a certain number of poor but respectable students, who belong to large families of straitened means, and are distinguished for their diligence and good behaviour.

In order to be admitted to the final examinations, the pupil must shew that he has acquitted himself well at the weekly ones, as well as at those at the end of each semestre. The Dean of the Faculty is obliged to pay special attention to this rule, or otherwise forfeit 20 florins to the general fund. Two students cannot be examined at the same time. The judgments of reception are *Satis*, *Bene*, or *Valde bene*. If two professors vote for the candidate's rejection, he must be examined over again at some future time, going through certain courses prescribed to him in the interval. If he decline this, he is not entitled to have his examination fees refunded to him; but if he submit to a second trial, he has nothing additional to pay. Not so, however, if he be rejected a second time: he must pay his fees afresh for a third examination. Nobody can be examined more than three times: a third rejection disqualifies the candidate from ever practising in Austria.

Previous to the admission of the candidate to examination, he must produce an account of two medical cases treated by himself, and also a report in legal medicine. These papers must be forwarded to the Dean, who communicates them to the examiners, and upon their approval the candidate is admitted to the final *rigorous* examinations.

The rigorous ordeals for the diploma are two in number. The first is an examination in anatomy, botany, natural history, physiology, general and special pathology of external and internal diseases, semeiology, and general therapeutics. The examiners are the Dean, the President of the Faculty, and the professors of anatomy, botany, natural history, physiology, and pathology.

For the second, the subjects are, chemistry, forensic medicine, ophthalmology, materia medica, art of prescribing, and clinical practice; and the examiners are the professors of chemistry, forensic medicine, ophthalmology, and materia medica, together with a physician unattached to the faculty. In Vienna, the latter person is the vice director, and in the provinces, some practising physician, not a professor.

The candidate may answer, as he pleases, either in Latin or in the vernacular.

Having passed these examinations, he is obliged to write a dissertation on a medical subject. He must also add some theses, which he has to defend publicly against three disputants—doctors of medicine or surgery. The Dean, and the President of the Faculty, as well as four professors, must attend the reading of the dissertation, and copies of it are to be distributed to all who may be present: the said dissertation and theses being written and defended in German, if the Dean grant leave; but this is not very usual, nor without some reasonable pretext.

The expenses of these final *rigorous* examinations are—for the first, thirty-five florins, five florins being paid to each examiner; for the second, sixty-three florins, nine florins to each examiner. The fee to the censorship, exercised by each of the professors in turn, is four florins fifty kreutzers; for the admission, &c. sixty-nine florins; to the president of the dissertation, twenty-seven florins: in all, 199 florins (nearly £20.)

If the candidate seek the diploma of doctor of surgery—1, He must be examined in anatomy, chemistry, materia medica, the art of prescribing, forensic medicine, ophthalmology, and the theory and practice of surgery; 2, He must perform two operations on the dead body publicly, and in presence of all the professional men and pupils who choose to be present. Previously to operating, he must give a history of the process which he is about to adopt, describe it, point out the different modes in which it may be performed, distinguish the several advantages and disadvantages of each mode, note the indication and contra-indication, shew how the instruments and bandages are to be employed, &c.: in short, he must act with all the care and attention he would use with the living.

If a doctor of surgery wish to obtain the degree of doctor of medicine, he must be examined—1, in botany, physiology, natural history, general and special pathology, therapeutics, and semeiology of internal diseases; 2, in practical matters relating to internal medicine. For both these examinations, the dissertation, and the admission fees, &c., the expenses are 114 florins, 30 kr. (about 11*l*. 10*s*.)

If a doctor of medicine wish to be admitted to surgery, he must be examined—1, in the theory and practice of surgery; 2, in the public test required of every candidate for the surgical diploma. The expenses are 110 florins (about 11*l*.)

Non-catholic candidates are admitted to degrees by dispensation only; but then there is no oath administered at variance with the religious tenets or observances of the parties.

### 2. ORGANIZATION OF THE PROFESSION.

We have now to give an account of the arrangements of the medical profession throughout the States. *The supreme direction of every thing that relates to the general organization of medical affairs, is committed to the Chancellory of the court of Austria.* In the provinces, it is entrusted to the provincial officers (*Landesstellen*), who, however, are obliged to have recourse to the Chancellory in all matters of importance. As all kinds of quarantine regulations, and the appointment of *cordons sanitaires*, rest with the Minister of War, the provincial magistracy have chiefly to attend to the epidemics which may visit their districts. They are enjoined to take all necessary measures to stifle epidemics at their birth, or at least to prevent their spread.

In every province of the hereditary states of Austria there is a medical man, charged with the supreme direction of sanitary arrangements. This is the *Landschafts-Proto-Medicus*, who is also a member of the council of state *(Sanitatsrath)*, with a deliberative voice in the provincial assemblies.

The director of medical studies in the University of Vienna is also the *Proto-Medicus* of the empire. His circle of activity is therefore, as may be conceived, extremely wide, for it comprehends the whole sanitary organization throughout every part of the Austrian monarchy. This officer is second only to the Chancellor, with whom he maintains close relations,—the latter demanding his advice on all arrangements connected with the public health. The appointment of the *Proto-Medici* of the provinces is in the hands of the Emperor; their salary is usually 1000 florins (about 100*l.* per ann.)

Every provincial government has a medical reporter attached to it, whose duty it is to attend at the meetings of the magistracy, to vote on all questions as one of that body, and to assist in the periodical statements required at head quarters, touching—1, the health of the local population and of their domestic animals; 2, the hospitals, their management, and the treatment therein adopted; 3, the apothecaries' shops; and 4, the conduct of the medical men who are in the service of government.

The provincial magistrates have the charge of the public health in their respective localities, and to them the district medical officers direct their reports on all such subjects; for example, as the rise and progress of epidemics, &c. On every occasion of adopting any new sanitory arrangement, they are obliged to take the opinions of the medical faculty of the province.

The district medical officers *(Kreisphysiker)* are appointed by the provincial authorities, with the consent of the *Proto-Medicus* of the province; and finally the government sanctions the appointment, if not otherwise advised.

In those towns which possess a university or a lyceum, the *Proto-Medici* are also directors of medical studies, presidents of the faculty, or the College of Physicians. But Vienna is excepted from this arrangement: there the *Proto-Medicus* of Lower Austria only exercises his jurisdiction beyond the capital. Those universities also are excepted which, like that of Pesth, possess at the same time a Director and a *Proto-Medicus.*

One part of the duties of the *Proto-Medici* is to exercise a political censorship on all works and articles in the journals connected with medicine; the authors are obliged to send their manuscripts to these officers previous to publication. Wherever there are both a *Proto-Medicus* and a *Director* in any city or town, it is the latter who acts as censor.

Among the other functions belonging to the *Proto-Medici* are—1, that of having an eye upon the different orders of practitioners, such as the oculists, dentists, apothecaries and midwives throughout the province; and, 2, that of superintending the hospitals, asylums, and prisons. Their qualifications for the post must comprehend an exact knowledge of the nature of the country, its inhabitants, and their habits of life —all with reference to the public health. He must offer suggestions to the government from time to time relative to the means of removing or destroying injurious influences; and his special duties embrace the noticing of every thing connected with ill-judged sites for building, the presence of marshes, bad water, the popular prejudices respecting the physical education of children, &c. He must also see that there is a sufficient supply of clever medical practitioners in each district, and that they be not too far asunder. Quacks, and charlatan practitioners of every sort, male and female, who have not duly qualified themselves by passing the proper ordeals, he is authorised to put down; and he must take care that nobody sells drugs except the regular apothecary, and that the latter offer for sale no emmenagogues, violent medicines, or poisons, unless when applied for through the recipe of a regular physician or surgeon. He has also to inspect the foundling and maternity hospitals.

On the occurrence of an epidemic, he must repair to the place, and take measures with the district practitioners for its subdual; and when it is over, he must draw up a full report of the circumstances of its rise, progress, nature, symptoms, &c. with such pathological and therapeutical observations as seem to be called for. The *pharmaciens*, and their establishments, are under his strict surveillance; with the injunction, that on the proper discharge of this duty, depends the safety of the subject from the pernicious consequences of bad drugs. When obliged to travel in the performance of his functions, he is paid his expenses and an indemnity. At the end of every year he is bound to send in a report to the government of the province, stating the general sanitory history of the annual period just elapsed: to this he adds a list of the births, marriages, and deaths, and of the numbers received into the hospitals, asylums, &c. with returns of the number cured, or who have died. He appends likewise an account of the atmospheric constitution of the year, and of all the phenomena which seem to affect the health of man and domestic animals: besides all the remarkable cases which have connexion with the province of medicine and surgery. All this is founded on the reports of the subordinate medical officers who have charge of the several districts.

The *Proto-Medicus* of a maritime province is by right a member of the sanitory council of that province, if he reside in the district.

The *Proto-Medicus* of Lower Austria has a most extensive range of duties. Among others, he visits once a month, and without previous notice, all the hospitals within his jurisdiction, including the lunatic asylum, &c.; and the results of his examination are transmitted to government. At the end of every year, he appends to his report a list of all the practitioners within his district, with their names, appointments, and the universities where they studied.

It is a repeated injunction in the ordinances, never to give the appointment of a physician to any one who has not served many years in a large hospital. Recently a law to that effect has been made; and in order to ensure its observance, it is customary, on the occurrence of a vacancy, to advertise it in the Vienna Gazette for several days previous to the final nomination by the court.

It is a strong recommendation to a candidate, that he shall have contributed articles to the "Medical Annals of Austria:" the titles of these articles must be set forth, and if they relate to epidemic or endemic diseases, to remarkable cases in pathology, to medical topography—or contain suggestions for the preservation or amelioration of the public health—they are the more favourably looked upon. The careful and successful practice of vaccination, *gratis*, on a great number of poor children, is also a passport to preferment.*

---

Our readers will hardly fail to be struck with the similarity of the above code, in its leading features, with that which we have often proposed and advocated in this Journal:—namely, the preliminary education—the extent of medical studies—the uniformity of education for physician and surgeon—the rigid examinations—the liberty to practise one or both branches of the profession—and, lastly, the supreme direction of medical polity vested in a central body; or, in other words, ONE FACULTY.

We are extremely gratified to learn that such a system has worked well—that it is not a mad Utopian scheme, engendered in the brains of medical reformers—and—that it has even found

* Med. Gazette, 22d Nov. 1834, as translated from a Belgian Journal.

favour in the eyes of our contemporary, the MEDICAL GAZETTE.

"If (says our contemporary) certain of our reformers in this country were really desirous of 'levelling upwards,' we could not propose to them a better standard, or model of imitation, than the Austrian system. It seems not unsuited, too, to become their *beau ideal* in another particular, inasmuch as it approaches their grand desideratum of a single faculty; and they find in it, moreover, a metropolitan body conferring degrees in the two great branches of the healing art. *We could ourselves, indeed, be even content with the adoption of some such system among us;* for it exhibits in actual practice that sort of projected alteration of which we have all along been the advocates. All the requisite studies are pursued in the metropolis—all the necessary qualifications, literary and professional, are there attained—and there, too, is (as we would have it) the final verdict of competency pronounced by a competent tribunal."

We were the first to make use of the expression, "levelling upwards"—and after mature reflection, we believe that the expression is as correct and intelligible as "levelling downwards," or any other kind of levelling. We were somewhat roughly handled by our contemporary for advocating any such system; but any soreness that we might have felt at the time, is amply soothed by the above avowal, that our then opponent would now "*be content with the adoption of some such system among us.*" We cannot flatter ourselves that any of *our* arguments worked this change in our contemporary; but we are very glad to find him advocating the same cause, whether induced so to do by the example of the Croat, or by his own reasonings on the subject.

It will not be imputed to us, that we are advocates of despotism, or that we would recommend a censorship of the medical press. But we confess that we should not object to the existence and duties of the PROTO-MEDICI, even in this land of liberty. We would not, indeed, tolerate the supervision of manuscripts before they go to press—though we suspect that such supervision might sometimes be useful, and that it sometimes takes place even here. We would not, however, object to some tribunal for the correction of quackish and scurrilous articles, after they are published—as well as for calling to account those individuals who act in an unprofessional manner towards their brethren, or the public at large.

---

